# Oxaliplatin, 5-fluorouracil and leucovorin (FOLFOX) as secondline therapy for patients with advanced urothelial cancer

**DOI:** 10.18632/oncotarget.10463

**Published:** 2016-07-07

**Authors:** Sheng Zhang, Hongxi Xue, Qiang Chen

**Affiliations:** ^1^ Medical Oncology, Fudan University Shanghai Cancer Center, Department of Oncology, Shanghai Medical College, Fudan University, Shanghai, China; ^2^ Rizhao City Hospital of Traditional Chinese Medicine, Rizhao, China; ^3^ Department of Clinical Biochemistry, School of Public Health, Taishan Medical University, Tai'an, China

**Keywords:** urothelial cancer, oxaliplatin, leucovorin, 5-fluorouracil, clinical trial

## Abstract

There is currently no standard treatment for metastatic urothelial cancer after failure of cisplatin-based therapy. The present retrospective study investigated the efficacy and safety of oxaliplatin plus 5-fluorouracil (5-FU) and leucovorin (LV) (FOLFOX) in locally advanced or metastatic urothelial cancer patients following cisplatin-based treatment. Thirty-three patients who had received one or two cisplatin-based regimens were treated with oxaliplatin (85 mg/m^2^) as a 2-h infusion on day 1, LV (200 mg/m^2^) as a 2-h infusion followed by bolus 5-FU (400 mg/m^2^) on day 1, or a 44-h continuous 5-FU (1,200 mg/m^2^) infusion. Patients were a mean of 67 years old with two involved organs. Metastases were mostly in the lung (43%), lymph nodes (51%) and liver (46%). Based on an intention-to-treat analysis, nine patients achieved a partial response, with an overall response rate of 27%. Eight (24%) patients had stable disease. Mean progression-free survival was 3 months and mean overall survival was 6.1 months. Toxicity was mild to moderate with grade 3 or 4 neutropenia, thrombocytopenia and neuropathy occurring in 5 (15%), 4 (12%) and 2 (6%) patients, respectively. This study demonstrated that oxaliplatin plus 5-FU/LV was a well-tolerated second-line regimen with moderate activity in patients pretreated with cisplatin-based therapeutics.

## INTRODUCTION

Urothelial cancer is the sixth most common cancer and accounts for more than 13,000 deaths annually [1]. Cisplatin-based chemotherapy is the standard of care for patients with metastatic or advanced disease [2], and about 50% of patients achieve an objective response. Methotrexate, vinblastine, doxorubicin and cisplatin (MVAC) or the combination of gemcitabine and cisplatin have become first-line treatment standards, based on results from randomized phase III trials [3, 4]. For patients who complete one of these cisplatin-based regimens, there is no standard second-line chemotherapy regimen. Under these circumstances, many single agents, including docetaxel, paclitaxel and pemetrexed, demonstrated moderate responses (between 10 and 20%) mainly in phase II studies, yet no drug has prolonged overall survival (OS) in randomized settings [5–8]. Vinflunine is a novel synthetic vinca alkaloid. In a phase III trial, cisplatin-refractory patients with metastatic or advanced urothelial cancer received vinflunine or best supportive care. Vinflunine did not confer an OS advantage across the trial population and consequently is not approved for patient use in the United States [9, 10]. However, OS advantage was demonstrated when only eligible patients were considered, and vinflunine was approved in Europe.

The lack of effective alternative treatment options for patients on cisplatin-based regimens emphasizes the need for novel therapeutics. In addition, impaired renal function in urothelial cancer patients compounds patient management challenges. Oxaliplatin has shown promising activity in metastatic urothelial patients in a few early trials [11]. When 37 adult cancer patients received oxaplatin in a phase I trial, no dose-limiting toxicities were observed in patients with creatine clearance ≥20ml/min. This suggests that oxaliplatin therapy is well tolerated by patients with mild to moderate renal dysfunction [12]. Oxaliplatin has also been used extensively with 5-fluorouracil (5-FU) and leucovorin (LV) in the treatment of colorectal cancer and others [13]. 5-FU was moderately active in the treatment of urothelial cancer patients in a pilot study [14]. On the basis of these encouraging data, we applied biweekly oxaliplatin therapy with LV and continuous 5-FU infusion in patients with urothelial cancer after failure of cisplatin-based therapy.

## RESULTS

### Patient characteristics and results

Thirty-three patients with locally advanced or metastatic urothelial cancer between January 2008 and March 2011 were selected for this study (Table 1). Mean patient age was 67 years (from 48 to 82 years) and two organs were involved on average (from one to five). Metastases were found mostly in the lung (43%), lymph nodes (51%) and liver (46%). Patients had received 1.4 prior cisplatin-based chemotherapy regimens on average (from one to two). Fifty-seven percent of patients responded to previous cisplatin-based chemotherapy treatments, while 43% did not.

**Table 1:** Patient Characteristics (N=33)

	No.	%
Male	28	85
Age, years		
Mean	67	
Range	48-82	
ECOG performance status		
0	15	45
1	15	45
2	3	10
Histology		
Transitional cell	29	88
Mixed	4	12
Site of primary tumor		
Bladder	29	88
Other(ureter or renal pelvis)	4	12
No. of metastatic sites involved		
1	10	30
2	11	33
3 or more	12	37
Visceral metastases		
Hepatic	14	42
Non-hepatic	13	40
Prior therapy		
Adjuvant chemotherapy	9	27
Chemotherapy for advanced disease	31	94
Radiation therapy	13	39
Prior radical cystectomy	15	45
Previous platinum response	18	55

Abbreviation:ECOG, Eastern Cooperative Oncology Group.

**Figure 1:**
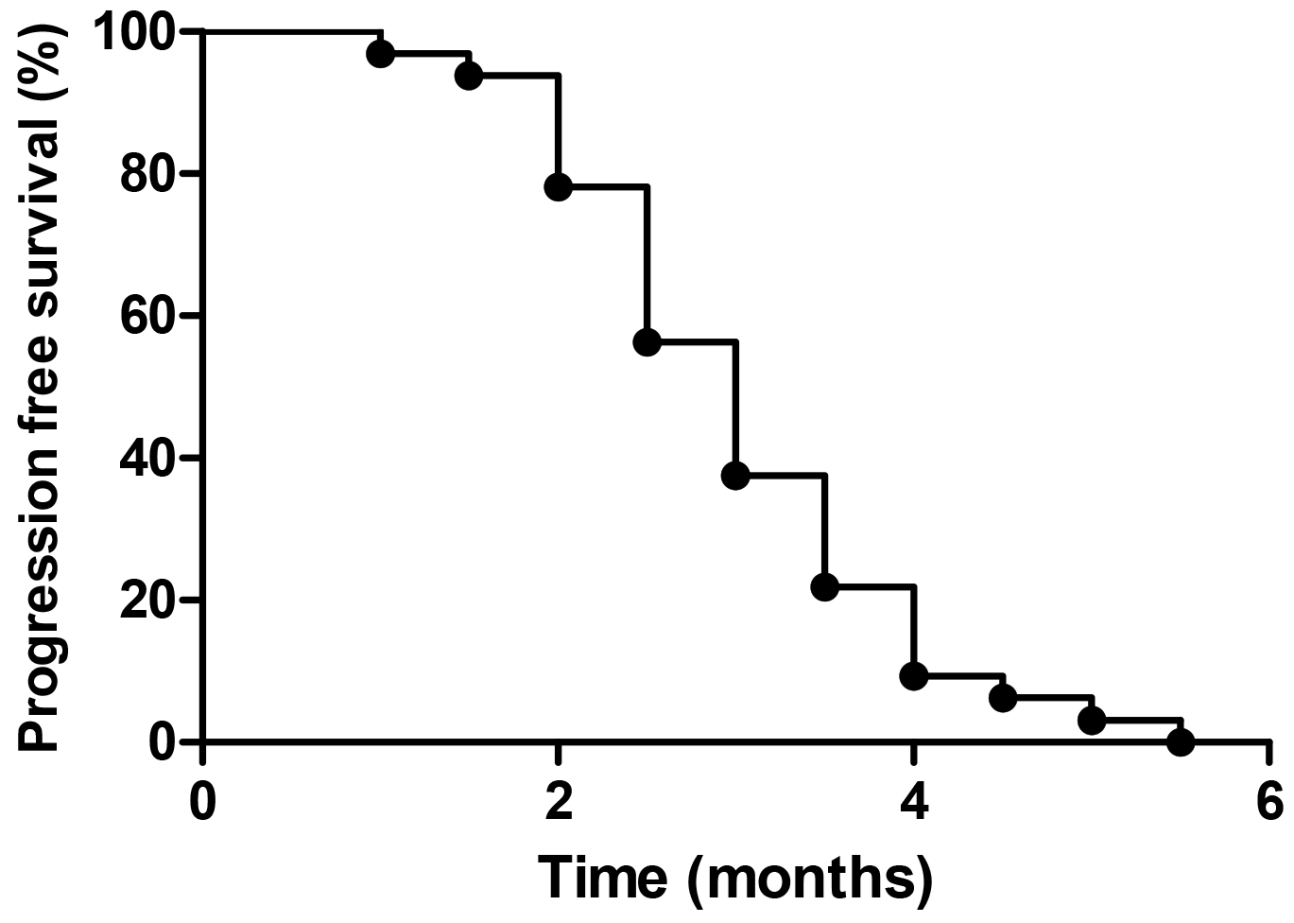
Profession-free survival among all patients

**Figure 2:**
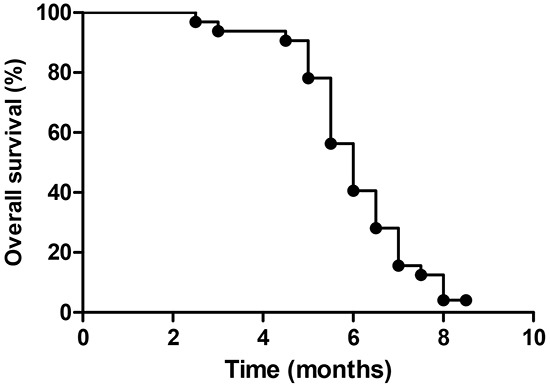
Overall survival among all patients

Of the 33 patients, none achieved CR and nine achieved PR during the study. The overall response rate was 27%, eight (24%) patients had SD and the CBR rate was 51% (Table 2). Mean PFS was three months (95% CI, 2.5 to 3.5, Figure 1), and mean OS was 6.1 months (95% CI, 4.2 to 8.3, Figure 2).

**Table 2:** Response to FOLOFX regimen (N=33)

	No. of patients	%
Response		
Complete response	0	0
Partial response	9	27
Stable disease	8	24
Overall response	9	27
Progressive disease	16	48
Clinical benefit rate	17	52

### Treatment regimen and toxicity

161 chemotherapy cycles were administered at an average of 3.5 cycles per patient (from 1–10). Toxicity was generally mild to moderate (Table 3), and the most common type of toxicity was hematological, with neutropenia and thrombocytopenia. Grade 3 or higher neutropenia, thrombocytopenia and neuropathy occurred in five (15%), four (12%), and two (6%) patients, respectively. Manageable gastrointestinal toxicity was reported, with grade 3 nausea and vomiting in only one patient. Nine (27%) patients needed at least one dose reduction, most commonly due to neutropenia (9%) or neuropathy (6%). No treatment-related deaths occurred.

**Table 3:** Toxicities of patients

Toxicity	Grade, n (%)			
	1	2	3	4
Anemia	13(39)	3(9)	0	0
Neutropenia	11(33)	4(12)	3(9)	2(6)
Thrombocytopenia	6(18)	3(9)	2(6)	2(6)
Nausea/vomiting	5(15)	4(12)	1(3)	0
Diarrhea	2(6)	1(3)	0	0
Fever	3(9)	1(3)	0	0
Neuropathy	6(18)	1(3)	2(6)	0

## DISCUSSION

Given that management of metastatic urothelial cancer remains a formidable challenge, a clear and unmet clinical need for novel therapies remains for patients resistant to cisplatin-based treatments [15–17]. In the present report, an oxaliplatin and 5-FU/LV (FOLFOX) treatment regimen was used in the management of urothelial cancer patients after failure of cisplatin-based treatment. With this regimen, patients with locally advanced or metastatic urothelial cancer, who had been pretreated with cisplatin-containing agents, achieved a 27% ORR and 51% CBR. Most previous studies evaluating multiple regimens achieved ORRs between 10 and 20% [16].

While our results were encouraging, our study might have been subject to certain biases common to retrospective studies, such as selection bias. Thus our results should be interpreted with caution. Our results appear consistent with those of several other small phase 2 studies [17, 18]. A phase 2 study of the folate antagonist pemetrexed showed a similar ORR of 27% [7]. Similarly, when 48 metastatic urothelial cancer patients were treated with nanoparticle albumin-bound paclitaxel at three-week intervals, a 28% ORR was achieved [19]. The single agent pazopanib was the first targeted agent to show significant clinical activity in previously treated urothelial cancer patients, with an ORR of 17% [20].

Rosenberg, *et al*. recently reported the results of an multicenter phase 2 study of atezolizumab in patients with locally advanced or metastatic urothelial carcinoma [21]. Compared with the historical ORR of 10%, atezolizumab therapy resulted in a significantly higher rate of 15%. Importantly, higher ORRs were achieved in individuals expressing higher programmed death-ligand 1 (PD-L1) levels. This finding must be confirmed in ongoing phase III trials.

The mean OS of 6.1 months was shorter than that of pemetrexed and docetaxel [7, 8]. It is possible that our study selected patients with relatively poor prognoses, as compared with other similar studies. Eighty-two percent of our patients had visceral metastases, and less than half had a PS of 0. Both poor PS and visceral (especially hepatic) metastases were independent negative prognostic factors in a phase 3 vinflunine study [9]. Our results were consistent with these findings.

Patients with advanced urothelial cancer after cisplatin-based treatment are generally older and more fragile, with possible poor PS and impaired renal function. For this reason, the majority of clinical trials in metastatic urothelial cancer are small phase 2 trials [22]. Our results provided an alternative second or third-line metastatic urothelial cancer therapy option, following failure of cisplatin-based treatment, and should be tested in prospective trials. Importantly, the oxaliplatin-based regimen described here does not require dose adjustments for patients with mild to moderate renal dysfunction, favoring this regimen over other choices under these conditions.

The safety profile of the described treatment regimen was acceptable. Grades 3 and 4 neutropenia, thrombocytopenia and neurotoxicity were experienced by 15, 12 and 6% of patients, respectively, and nine (27%) patients required dose reductions. Additional adverse responses included anemia, nausea and vomiting, diarrhea and fever, and no treatment-related deaths occurred.

In conclusion, in patients for whom cisplatin-based treatment has failed, oxaliplatin, LV and 5-FU in combination are well tolerated and demonstrate clinically meaningful activity. Treatment efficacy should be further confirmed in prospective, and ideally randomized, clinical trials.

## METHODS AND MATERIALS

### Patients

This study was a single-institution retrospective analysis within the Department of Internal Medicine of Shanghai Cancer Hospital, China. The study was approved by the institutional review board, and informed patient consent was waved because of the retrospective nature of the study.

Eligibility criteria included: (1) age 18 years or older, Eastern Cooperative Oncology Group (ECOG) performance status (PS) 0-2 and a life expectancy of more than 12 weeks; (2) histologically-diagnosed, measurable locally advanced or metastatic transitional carcinoma of the urinary bladder urothelium or upper urinary tract; (3) documented disease progression after first or second-line cisplatin-based treatment; (4) no prior treatment with 5-FU infusion and/or oxaliplatin therapy; (5) and adequate liver, renal (calculated creatinine clearance ≥30ml/min by the Cockcroft-Gault formula), medullary and cardiac functions. Patients previously treated with radiotherapy were eligible for the study, provided that measurable disease existed outside the radiation field. Patients with brain metastases were eligible provided that they had received cranial irradiation with clinical and radiological improvement of their central nervous system disease. Patients were excluded from the study if they had secondary malignancy (except for carcinoma of the skin) and preexisting motor or sensory neurotoxicity grade ≥2, according to the Common Terminology Criteria for Adverse Events 3.0 (CTCAE 3.0) scale (intolerable paresthesias and/or marked motor loss). Fertile patients without the use of adequate contraceptive measures and pregnant or breast-feeding women were ineligible for the study. Patients with active infection or other serious underlying medical conditions that would impair their ability to receive the treatment or those without appropriate medical files were also excluded from the study.

### Study design

Oxaliplatin was administered at 85 mg/m^2^ in 5% glucose as a 2-h infusion on day 1, LV 200 mg/m^2^ as a 2-h infusion followed by bolus 5-FU 400 mg/m^2^ on day 1, and a 44-h infusion of 5-FU 1,200 mg/m^2^. Treatment was repeated every two weeks. Patients were evaluated for response usually every eight weeks. Patients were premedicated with antiemetics, including 5-hydroxytryptamine-3 receptor antagonists and corticosteroids. Hematopoietic growth factors and transfusion were allowed. Treatment was continued until disease progression, unacceptable toxicity, death or withdrawal of informed consent.

Oxaliplatin and 5-FU doses were reduced by 25% in patients who experienced dose-limiting toxicity (DLT), defined as grade 4 neutropenia lasting for more than seven days, febrile neutropenia, grade 4 thrombocytopenia, grade 3 thrombocytopenia associated with bleeding or grades 3–4 non-hematological toxicities (except alopecia and neuropathy). In the presence of grade 3/4 neurotoxicity, treatment was delayed until recovery to toxicity grade ≤1 (no longer than 14 days). These patients continued to receive reduced doses in subsequent cycles for the remainder of the study. Two dose reductions were allowed. These patients were discontinued from the study if there was evidence of disease progression, presence of unacceptable toxicity, interruption of treatment for more than two weeks, withdrawal of informed consent or if a third dose reduction was required.

### Study assessment

Pretreatment evaluation included a complete medical history and physical examination, hematological and biochemical profiles, electrocardiography (ECG), and computed scan of the chest, abdomen and pelvis. During the treatment period, complete blood counts were performed weekly or every two days in cases of grade 3/4 neutropenia or thrombocytopenia until hematological recovery occurred. Efficacy was evaluated in patients who received at least two chemotherapy cycles. Patients were evaluated for response according to the National Cancer Institute's (US) response evaluation criteria in solid tumors (RECIST). Complete response (CR) was defined as the disappearance of all known lesions and normalization of tumor marker levels for at least four weeks. Partial response (PR) was defined as a reduction in the sum of all measurable lesions by at least 30% for at least four weeks. Progressive disease (PD) was defined as an increase in the sum of all measurable lesions by >20% or the appearance of a new lesion, and stable disease (SD) was defined as neither CR, PR nor PD. Overall response rate (ORR) was defined as the sum of CR and PR rates. Clinical benefit rate (CBR) was defined as the sum of CR, PR and long SD rates. In patients with tumor response or stable disease, the treatment was continued for up to 8–12 cycles; thereafter, maintenance therapy was based on the clinician's decision. After completion of the treatment period, the patients were followed up every 1.5 months. All adverse events were graded according to the National Cancer Institute's Common Toxicity Criteria, version 3.0.

### Statistical analysis

All statistical analyses were carried out on an intention-to treat basis with SPSS 17.0 software (Chicago, Illinois, USA). Progression-free survival (PFS) was calculated for all assessable patients as the time from inclusion to disease progression or death from any cause. Overall survival (OS) was calculated for all patients from the date of inclusion until death. PFS and OS were computed using the Kaplan–Meier method.
